# Plasma PARK7 and NDKA in acute stroke: methodological issues regarding diagnostic value

**DOI:** 10.2144/fsoa-2019-0078

**Published:** 2019-10-30

**Authors:** Siamak Sabour

**Affiliations:** 1 Department of Clinical Epidemiology, School of Health & Safety, Shahid Beheshti University of Medical Sciences, Tehran, Iran; 2 Department of Epidemiology, Safety Promotions & Injury Prevention Research Centre, Shahid Beheshti University of Medical Sciences, Tehran, Iran

**Keywords:** acute stroke, biomarker, diagnostic value, hemorrhagic stroke, ischemic stroke, NDKA, PARK7

Letter in response to: Tulantched DS, Min Z, Feng WX. Comparison of plasma PARK7 and NDKA diagnostic value in acute stroke. *Future Sci OA* 5(5), FSO375 (2019) [1].

I read a recently published article by Tulantched *et al*. in *Future Science OA*. The purpose of this study was to compare the diagnostic value of plasma PARK7 and NDKA in early diagnosis of acute stroke and evaluate the validated diagnostic values of PARK7 and NDKA in an independent patient cohort. The authors state that the expression of PARK7 (area under the curve = 0.89) in acute stroke patients was more significant than in controls, relative to the NDKA expression (area under the curve = 0.462); p < 0.05. Finally, they concluded that even though both markers cannot differentiate stroke etiologies (ischemic or hemorrhagic), plasma PARK7 has better diagnostic value than NDKA for early diagnosis of stroke [1]. The authors conclusions and comments are further summarized briefly in the highlighted sentences here:

- PARK7 seems to be the most accurate biomarker for stroke diagnoses and might be involved in triage of patients with acute stroke.
- Both markers PARK7 and NDKA have limitations; they are similarly expressed in ischemic stroke and hemorrhagic stroke, so they are not able to specify stroke type.
- The study sample size is small, but further large studies are ongoing in this field.
- More studies including healthy volunteers or patients with other diagnoses initially suspected as stroke are needed to specify the usefulness of both biomarkers PARK7 and NDKA.

This letter focuses on some important factors of diagnosis and biomarkers. Although it is crucial to understand that the above conclusions and comments of the authors are highly important, we would like to add methodological comments to more clearly clarify conclusion of the study. Diagnosis is the identification of the nature and cause of a certain phenomenon. The information required for diagnosis is typically collected from a history and physical examination of the person seeking medical care. Often, one or more diagnostic procedures, such as medical tests, are also done during the process. It should be noted that diagnostic value and early diagnostic value of a test are included in the assessment of both validity (accuracy) and reliability (precision) of the test.

Reliability (precision, repeatability) and validity (accuracy) are two completely different methodologic issues – that is a point that it is crucial to appreciate. Estimates to assess validity (accuracy) of a diagnostic test include false-positive and false-negative; sensitivity; specificity; positive predictive value; negative predictive value; likelihood ratio positive (LR+; ranging from 1 to infinity; the higher the LR+, the more accurate the test); and likelihood ratio negative (LR-; ranging from 0 to 1; the lower the LR-, the more accurate the test). These have nothing to do with reliability [2–5]. Reliability (precision) is a different methodologic issue and should be assessed using appropriate tests. For qualitative variables, the weighted κ can be applied with caution. Regarding quantitative variables, the intraclass correlation coefficient (ICCC) and Bland–Altman plot are among the well-known approaches. Second, when the authors report area under the receiver operating characteristic curve, they are actually reporting the validity (accuracy, discrimination) of a model instead of a diagnostic test. Therefore, it is crucial to not confuse a single diagnostic test with a diagnostic model, because assessing validity of the mentioned concepts are completely different. Finally, for clinical purposes, reporting diagnostic added value of the Plasma PARK7 and NDKA to interview and physical examinations (symptoms and signs) is much more important than simply reporting receiver operating characteristic for the mentioned biomarkers [2–7].

In clinical studies, distinguishing between concepts of ‘diagnosis’ and ‘early diagnosis’ is essential. The differentiation of these concepts should explain clearly and, in some cases, ‘diagnostic value’ and ‘early diagnostic value’ should not be used interchangeably. An early detection refers to screening, and the purpose of a screening test is to detect early disease or risk factors in the public population or healthy individuals, while a diagnostic test is a confirmatory test that establishes the presence or absence of disease in symptomatic individuals. In practice, early detection should help to prevent or reduce the adverse effects of expected outcome. So, clarified definition and determination of properties of diagnosis/early diagnosis, such as the target population, result thresholds, exact phase and period of time for investigation is very important.

To make our comments brief, we can highlight the below mentioned conclusions.

First, for clinical purposes, reporting diagnostic added value of the plasma PARK7 and NDKA to interview and physical examinations (symptones and signs) is much more important than simply reporting ROC for the mentioned biomarkers [2–7]. Second, in practice, early detection should help to prevent or reduce the adverse effects of expected outcome. So, clarified definition and termination of properties of diagnosis/early diagnosis, such as the target population, result thresholds, exact phase and period of time for investigation is very important.
